# A Variation in FGF14 Is Associated with Downbeat Nystagmus in a Genome-Wide Association Study

**DOI:** 10.1007/s12311-020-01113-x

**Published:** 2020-03-10

**Authors:** Michael Strupp, Stephan Maul, Bettina Konte, Annette M. Hartmann, Ina Giegling, Sophia Wollenteit, Katharina Feil, Dan Rujescu

**Affiliations:** 1 Department of Neurology and German Center for Vertigo and Balance Disorders, Ludwig Maximilians University, Munich Campus Großhadern, Marchioninistr. 15, 81377 Munich, Germany; 2 Department of Psychiatry, Psychotherapy and Psychosomatics, Martin-Luther-University Halle-Wittenberg, Julius-Kühn-Str. 7, 06112 Halle, Germany

**Keywords:** Downbeat nystagmus, Genome-wide association study, Fibroblast growth factor 14 (FGF14), Dihydrofolate reductase (DHFR), Cerebellum

## Abstract

Downbeat nystagmus (DBN) is a frequent form of acquired persisting central fixation nystagmus, often associated with other cerebellar ocular signs, such as saccadic smooth pursuit or gaze-holding deficits. Despite its distinct clinical features, the underlying etiology of DBN often remains unclear. Therefore, a genome-wide association study (GWAS) was conducted in 106 patients and 2609 healthy controls of European ancestry to identify genetic variants associated with DBN. A genome-wide significant association (*p* < 5 × 10^−8^) with DBN was found for a variation on chromosome 13 located within the fibroblast growth factor 14 gene (FGF14). FGF14 is expressed in Purkinje cells (PCs) and a reduction leads to a decreased spontaneous firing rate and excitability of PCs, compatible with the pathophysiology of DBN. In addition, mutations in the FGF14 gene cause spinocerebellar ataxia type 27. Suggestive associations (*p* < 1 × 10^−05^) could be detected for 15 additional LD-independent loci, one of which is also located in the FGF14 gene. An association of a region containing the dihydrofolate reductase (DHFR) and MutS Homolog 3 (MSH3) genes on chromosome 5 was slightly below the genome-wide significance threshold. DHFR is relevant for neuronal regulation, and a dysfunction is known to induce cerebellar damage. Among the remaining twelve suggestive associations, four genes (MAST4, TPPP, FTMT, and IDS) seem to be involved in cerebral pathological processes. Thus, this GWAS analysis has identified a potential genetic contribution to idiopathic DBN, including suggestive associations to several genes involved in postulated pathological mechanisms of DBN (i.e., impaired function of cerebellar PCs).

## Introduction

There is a wide range of hereditary and non-hereditary cerebellar ataxias (CA) with variable clinical symptoms, usually characterized by gait and limb ataxia, dysarthria, and oculomotor disorders, such as gaze-evoked nystagmus or saccadic smooth pursuit [1]. Although many genetic causes of CA have been discovered in recent years [2], several cases remain idiopathic. Unknown genetic factors and genetic susceptibility factors have been suggested as contributing to the degeneration of the cerebellum [2, 3].

Although downbeat nystagmus (DBN) is the most common involuntary fixation nystagmus [4], it is a very rare sign. DBN is often associated with other cerebellar ocular signs such as saccadic smooth pursuit or gaze-holding deficits (for ref. see [5, 6]). The most common symptoms of DBN are unsteadiness of gait (89% idiopathic DBN vs. 81% secondary DBN) and oscillopsia (44% vs. 38%) [4]. It is most often caused by a bilateral hypofunction of the flocculus or paraflocculus [7, 8] which – due to an impaired function of the Purkinje cells (PCs) – causes a disinhibition of superior vestibular nuclei neurons, leading to a slow upward drift of the eyes and a quick downward saccade [9]. Several theories concerning the pathophysiology of DBN have been proposed: (A) an asymmetry of peripheral vestibular input [10], (B) a central imbalance in the vertical vestibulo-ocular system [9, 11], (C) an imbalance of the smooth pursuit system [7, 12], and (D) a mismatch of the coordinate system of burst generator and the vertical generator [13].

In many cases, the etiology remains unclear (so-called idiopathic DBN: 38%); in other cases, an underlying structural pathology can be found, such as degenerative disorders of the cerebellum (20%), vascular lesions (9%), and malformations (7%) [4]. DBN is also frequently found in patients with spinocerebellar ataxia type 6 (SCA6) as well as in episodic ataxia type 2 (EA2) [14], whereas DBN was not found in other genetic CA such as SCA1, SCA2, SCA3, and SCA 31 [15–17]. All in all, despite its high prevalence and its characteristic clinical features which allow diagnosis, the underlying etiology of DBN often remains unclear.

Therefore, we used a different approach and performed a genome-wide association study (GWAS) in patients with idiopathic DBN in order to identify genes which might be associated with the disease and to allow a further evaluation of the pathophysiology and etiology of DBN.

## Material and Methods

### Patients

Patients of European descent were recruited in the German Center for Vertigo and Balance Disorders and the Department of Neurology at the Ludwig Maximilians University (LMU), Munich from 2012 to 2017. The detailed medical history, medication, and harmful substance abuse of each participant were assessed using a semi-structured interview. In addition, a detailed family history of first-degree relatives was collected, focusing in particular on neurological genetic disorders and DBN. None of the DBN patients reported cerebellar disorders in the parent generation. All patients underwent an extensive neurological examination and cerebral imaging by MRI or CT. Any evidence of symptomatic DBN led to exclusion from the study (see 2.1.2).

#### Inclusion Criteria

All patients included showed DBN, which has been defined as downwardly beating fixation nystagmus in primary position with an increase of the intensity of the nystagmus during lateral and downward gaze. The patients included in this study can be divided into two subgroups: (1) idiopathic DBN in association with other cerebellar oculomotor disorders (85 patients); and (2) idiopathic DBN in association with other cerebellar disorders such as cerebellar ataxia or with cerebellar atrophy (21 patients).

#### Exclusion Criteria

Subjects with any evidence of symptomatic DBN were excluded. This included cerebellar/brainstem infarction or hemorrhage; cerebellar tumor; evident neurodegenerative cerebral disorders/syndromes (e.g., multiple system atrophy); inflammatory, infectious, and immune-mediated cerebellar damage; toxic and nutritional cerebellar damage (e.g., due to alcohol); paraneoplastic cerebellar degeneration; or other infratentorial structural lesions.

### Healthy Volunteers (PAGES)

Healthy volunteers were recruited to the PAGES (Phenomics and Genomics Sample) sample from the Munich greater area and were of German descent. PAGES consists of approximately 3000 healthy individuals with a detailed medical, neurological, and psychiatric history of the participants themselves, and their first-degree relatives who were assessed using semi-structured and comprehensive interviews including the Structured Clinical Interview for DSM-IV (SCID I, II) [18, 19] and the Family History Assessment Module [20]. Individuals suffering from psychiatric or neurological diseases as well as subjects with CNS impairment or a self-reported history of DBN were excluded.

### Genotyping

Genomic DNA was extracted from whole blood using the QIAamp DNA Maxi Kit (Qiagen), according to the manufacturer’s instructions and dissolved in nuclease-free water. The concentration of genomic DNA was measured using Picogreen (Molecular Probes) and adjusted to 50 ng/μl. Samples were genotyped on different platforms.

### Genome-Wide Association Analysis

Genotyping and association analysis were performed as previously described [21].

#### Overview

Genotypes of the different platforms were imputed in seven batches and combined into one large dataset. Quality control and imputation of batch 1 (Human610-Quad [22], Human660W-Quad [23]), batch 2 (HumanHap300 [24]), and batch 3 (Affymetrix 6.0 [25]) were performed in the framework of a schizophrenia meta-analysis conducted by the Psychiatric Genomics Consortium (PGC) [26]. Batches 4 (HumanHap300 [27]), 5 (Illumina HumanOmniExpress-12 [28]), 6 (Illumina Omni1-Quad [29]), and 7 (HumanOmniExpress-24) were processed following quality control and imputation protocols, used by the PGC. Datasets were combined to get a sufficient number of controls. For GWAS analysis, selected patients and controls were extracted from the combined dataset after global quality control [21].

#### Global Quality Control

PLINK 1.9 [30] was used for global quality control of the genotype data. A pre-QC filtered SNV (single nucleotide variant) set (missingness *<* 0*.*05) was used to exclude subjects with mismatches between reported and estimated gender and samples with call rates below a chip-specific threshold. Sample call rate thresholds differed slightly between chips to account for smaller sample sizes (96–99%). After subject removal, SNV quality was assessed, and variations were excluded based on the following criteria: SNV call rate < 99%, deviations from Hardy-Weinberg equilibrium in controls (*p* ≤ 10^−6^) or cases (*p* ≤ 10^−10^) and SNVs with call rate differences ≥ 0*.*02 between cases and controls. Also, x-chromosomal markers with a haploid heterozygosity rate > 2%, missingness ≥ 0*.*05, or HWE *p* ≤ 10^−6^ in females were removed. A more stringent quality-controlled (MAF ≥ 0*.*05, HWE *p* ≥ 0*.*05, call rate ≥ 0*.*99) and LD-pruned autosomal marker set was used for cryptic relatedness, heterozygosity deviation and population stratification analyses. The marker set was pruned with PLINK’s indep-pairwise command using *r*^2^ = 0*.*2, a window size of 1500 and shifting the window 150 SNVs at each step. Additionally, several high LD regions were excluded as the extended MHC region. One subject of each pair with π̂ > 0*.*1875 was removed; cases were generally preferred over controls. As sample contamination results in an increase of heterozygote calls and thereby an overestimation of cryptic relatedness, individuals showing a heterozygosity deviation with |*Fhet*| ≥ 0*.*2 were excluded. In addition, the number of π̂-values > 0*.*05 per individual and the distribution of π̂ means were used to check for outliers and possible sample contamination. Population stratification analysis was done with EIGENSTRAT [31]. SNV loadings were checked for normality, and the derived principal components were used to identify and remove outlying samples. Known duplicates on different chips were checked for concordance, keeping the sample of higher quality (i.e., sample call rate and overall chip quality). Both subjects were excluded if the concordance rate was lower than 99%.

#### Pre-Phasing and Imputation

Each batch was pre-phased with SHAPEIT [32] and imputed separately on the 1000 Genomes reference panel phase 1 version 3 macGT1 (https://mathgen.stats.ox.ac.uk/impute/data_download_1000G_phase1_integrated.html) in chunks of 3 Mb using IMPUTE2 [33]. Chromosome X imputation was performed separately for males and females. After imputation, the seven batches were combined by retaining markers with INFO values ≥ 0*.*6 in every batch and the combined set. Additionally, all markers with allele frequency differences > 0.1 between any of the batches were excluded. Checks for cryptic relatedness, heterozygosity deviation, and population stratification on the combined set were performed, as described before, using the same exclusion criteria on a stringent-thresholded marker set (INFO > 0.8, missingness < 1%, MAF > 0.05) for calling best guess genotypes with uncertainty ≤ 0*.*1.

#### GWAS Analysis

**Fig. 1:**
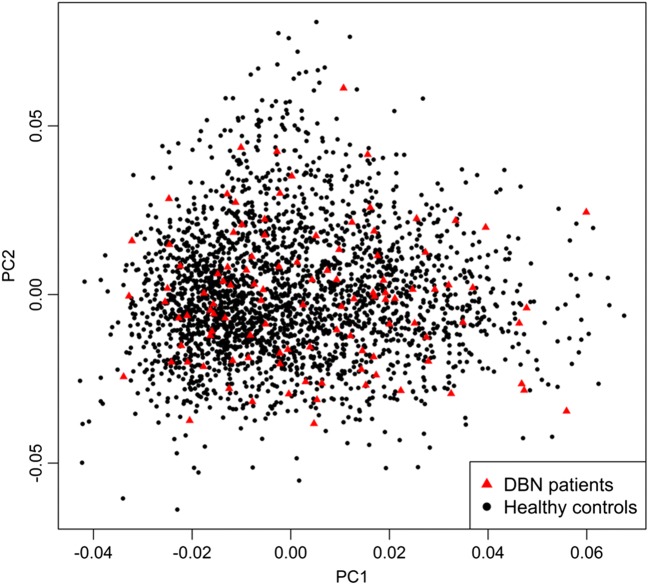
Scatterplot of the first two principal components (PC) derived by EIGENSTRAT

The final mega dataset was comprised of 4575 individuals, including 107 DBN patients and 2618 healthy controls appropriate for association analysis spread across 6 batches (Table 1). The principal components for genome-wide association analysis were derived by EIGENSTRAT [31]; 1 outlying case and 9 controls were removed. Tracy-Widom statistics identified PC1 and PC2 as relevant (Fig. 1). As DBN cases were typed on one platform, controls of the six batches were used for batch effect detection. Any marker showing deviations between any of the batches was excluded (logistic regression corrected for PC1 and PC2, *p* < 0.001). After exclusion of 116,387 variants, the final dataset was comprised of 7,759,885 markers.

**Table 1:** Batch distribution of cases and controls

	Post-imputation	GWAS	
Batch	*N*	*N* controls	*N* cases
1	319	0	0
2	709	262	0
3	954	925	0
4	287	99	0
5	607	578	0
6	352	267	0
7	1347	478	106
Combined	4575	2609	106

**Fig. 2:**
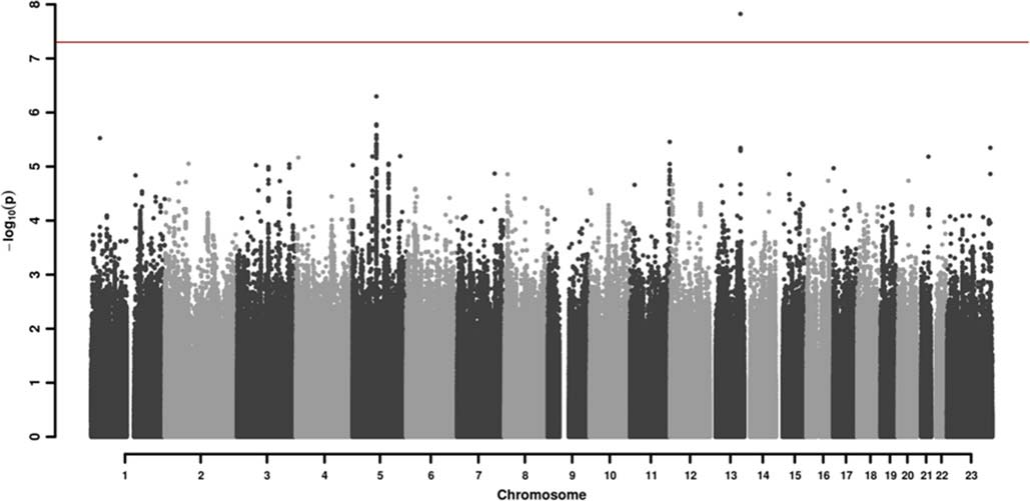
Manhattan plot of the genome-wide association analysis of 106 DBN cases and 2609 controls. The x-axis shows the chromosomal position, and the y-axis shows the significance of association (−log_10_(*p*)). The red line shows the genome-wide significance level (5 × 10^−8^)

**Fig. 3:**
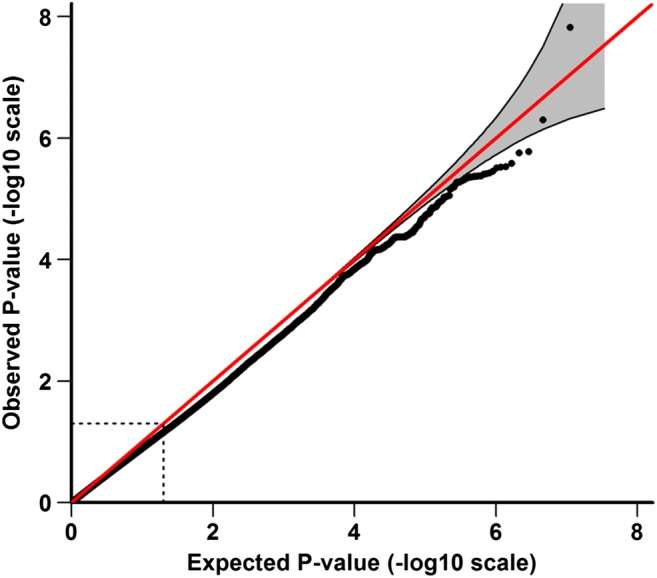
Quantile-quantile plot of GWAS analysis. The area shaded in gray indicates the 95% confidence interval under the null

GWAS association testing of approximately 8 million variants (INFO ≥ 0.6, MAF ≥ 0.01 in cases and controls) using 106 patients with DBN and 2609 healthy subjects was conducted in PLINK 1.9 [30], applying an additive logistic regression model corrected for age, sex, PC1, and PC2. Quantile-quantile and Manhattan plots are shown (Figs. 2 and 3). The genomic inflation factor was 1, thus showing no sign of global inflation due to batch effects or population stratification. Results were clumped with PLINK to derive LD-independent index SNVs (3000 kb, *p1* = 5 × 10^−08^, *p2* = 1 × 10^−4^, *r*^*2*^ < 0.1).

## Results

One hundred and six patients with idiopathic DBN (46 females, 43%) and 2609 healthy controls (1402 females, 54%) were included in the analysis. The mean age of the patients was 70.09 ± 10.1 years; the mean age of the controls was 47.41 ± 16.6 years. Among approximately 8 million variants tested for association, we identified one genome-wide associated SNV (*p* < 5 × 10^−8^) located in the fibroblast growth factor 14 gene (FGF14) mapping to chromosome 13q33.1 (Figs. 2 and 3, Table 2).

**Table 2:** LD-independent SNV associations for DBN

Chr. (region)	SNV	A1/2	Info	Frq_con_	Frq_case_	OR (95% CI)	*p* value	Gene (dist.)
Chr. 13 102,703,679–103,052,397	rs72665334	T/C	0.720	0.074	0.189	4.30 (2.60–7.13)	**1.50E-08**	FGF14 (0)
	chr13_103023008_D	TAAC/−	0.956	0.945	0.840	0.31 (0.19–0.51)	4.52E-06	
Chr. 1 30,238,075–30,238,075	rs147830437	T/C	0.928	0.223	0.343	2.36 (1.65–3.38)	2.98E-06	LINC01648 (246982)
Chr. 2 79,338,772–79,338,772	rs140798366	A/G	0.725	0.022	0.065	7.25 (3.03–17.38)	8.85E-06	REG1A (8812)
Chr. 3 63,595,461–63,595,461	rs113612577	T/C	0.872	0.014	0.053	6.38 (2.81–14.5)	9.44E-06	SYNPR (0)
Chr. 3 175,540,277–175,540,538	rs113420566	C/G	0.732	0.954	0.908	0.21 (0.11–0.42)	9.06E-06	NAALADL2 (16849)
Chr. 4 8,190,987–8,190,987	rs148323050	T/C	0.666	0.013	0.046	12.49 (4.16–37.53)	6.82E-06	SH3TC1 (10073)
Chr. 5 662,547–676,768	rs12516404	A/G	0.814	0.133	0.244	2.57 (1.69–3.91)	9.45E-06	TPPP (0)
Chr. 5 66,364,063–66,455,108	rs74495954	T/C	0.998	0.985	0.953	0.15 (0.06–0.34)	6.51E-06	MAST4 (0)
Chr. 5 79,902,336–80,171,134	rs245100	A/G	0.966	0.747	0.557	0.45 (0.32–0.61)	5.03E-07	DHFR (0) MSH3 (0)
	rs33003	A/G	0.985	0.345	0.170	0.39 (0.26–0.58)	3.87E-06	
Chr. 5 120,836,941–120,894,696	rs111326090	C/G	1.025	0.814	0.680	0.47 (0.33–0.65)	8.85E-06	FTMT (303199)
Chr. 5 160,104,068–160,104,068	rs151003482	A/G	0.947	0.017	0.064	6.04 (2.77–13.21)	6.43E-06	ATP10B (0)
Chr. 11 133,618,819–133,657,216	rs34790786	A/G	0.959	0.816	0.701	0.43 (0.30–0.61)	3.49E-06	SPATA19 (53301)
Chr. 21 39,418,784–39,541,479	rs138188997	C/G	0.754	0.972	0.931	0.17 (0.08–0.37)	6.56E-06	DSCR4 (7529)
Chr. X 148,510,680–148,532,290	rs187733794	T/C	1.001	0.020	0.078	4.89 (2.48–9.65)	4.49E-06	IDS (28005)

Genome-wide significant association is shown on top (*p* value in bold); otherwise, SNVs are sorted by genomic position according to UCSC hg19/NCBI build 37. The deletion variant is given in the form “chromosome_position_deletion.” Column A12 contains the SNV alleles, with the first allele (A1) depicting the reference allele for the frequency (Frq) and odds ratio (OR) of A1. *CI* is the confidence interval, gene is the next gene, dist. is the distance to index SNV in bp. Chromosome (Chr.) and position denote the associated region surrounding the index SNV containing SNVs in LD (*r*^*2*^ > 0.6) with the index SNV. Two LD-independent regions were merged on account of a distance below 250 kb (shaded in gray)

In addition, 15 LD-independent loci with suggestive evidence of association (*p* < 1 × 10^−5^) were identified. On chromosome 13q33.1 and chromosome 5q14.1, two regions each were merged on account of physically mapping next to each other with a distance below 250 kb, resulting in 14 physically and LD-independent regions (Table 2).

**Fig. 4:**
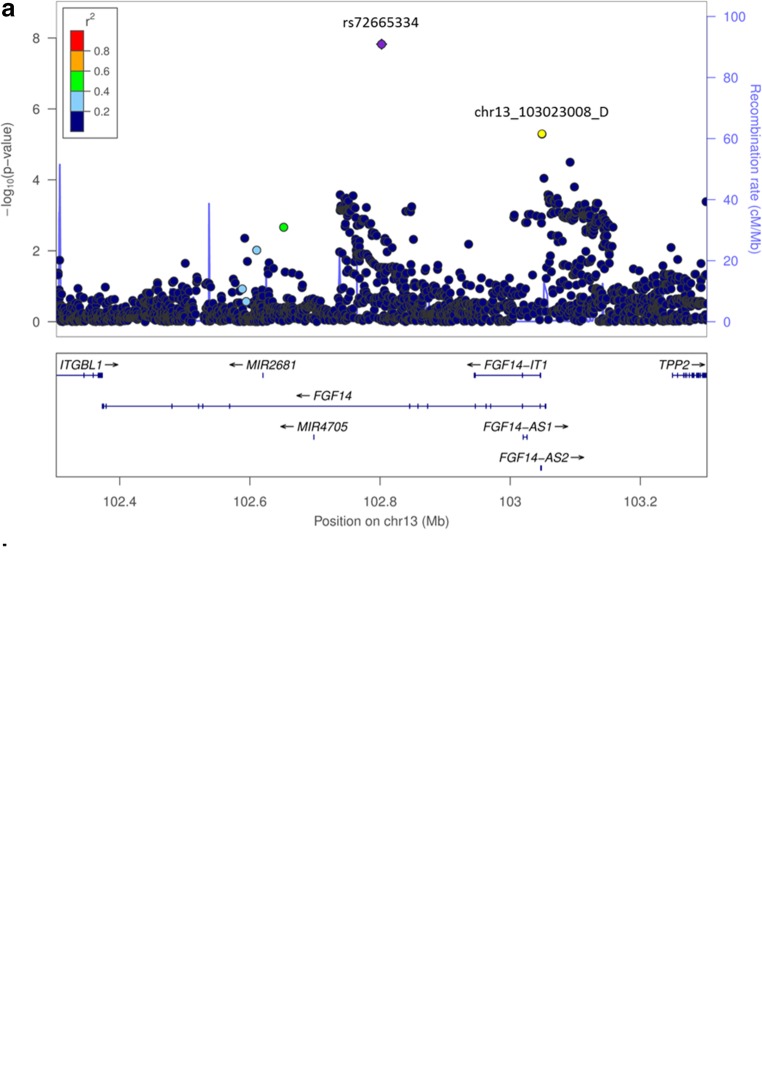
Regional association plots for loci associated with DBN. In order to highlight the statistical strength of the association in the context of the surrounding markers, gene annotations and estimated recombination rates (NCBI build 37) of the SNPs in the specific regions are plotted against their corresponding *p* values (as −log_10_ values, left-hand y-axis). A purple diamond represents the SNP with the highest association signal in each locus. All other SNPs are represented as single dots, where dot colors indicate the LD with the associated SNP. Color coding represents the *r*^*2*^ value, and respective categories are shown on the upper left hand side. Estimated recombination rates (cM/Mb) are plotted to reflect the local LD structure surrounding the associated SNP and are shown as vertical light blue lines, marked on the right-hand y-axis. Genes in the region are displayed below. The orientation of the genes is indicated by arrows. **a** Regional association plot of the FGF14 variations on chromosome 13q33.1 associated with DBN. The genome-wide–associated SNP (rs72665334, purple diamond) is localized in intron 9, the suggestive hit chr13_103023008_D (yellow circle) in intron 1 of the fibroblast growth factor 14 gene (FGF14). LD structure refers to the genome-wide associated variant rs72665334. **b** Regional association plot of the merged region on chromosome 5 containing the overlapping genes DHFR and MSH3 and the respective hits (rs245100, purple; rs33003, yellow). LD structure refers to the associated variant localized in DHFR (rs245100)

The merged region on chromosome 13 contained parts of the FGF14 gene, with the genome-wide associated variant rs72665334 (*p* = 1.50 × 10^−8^) being localized in intron 9 and the suggestive variant chr13_103023008_D (*p* = 4.52 × 10^−5^) in intron 1 of the gene (Fig. 4a). These hits showed isolated signals and should therefore be interpreted with caution.

The most promising region with suggestive evidence for association is localized on chromosome 5q14.1 containing two genes with a head-to-head overlap of their respective exons 1: dihydrofolate reductase (DHFR) and mutS (Mutator S) protein homolog 3 (MSH3). Two LD-independent hits are present in this merged region. Variant rs245100 (*p* = 5.03 × 10^−07^) is located in intron 4 of the DHFR gene, and rs33003 (*p* = 3,87 × 10^−06^) is located in intron 23 of the MSH3 gene (Fig. 4b).

Another four regions with suggestive evidence were also located on chromosome 5. Apart from one region on chromosome 5q23.1 in which the index SNV was located approximately 300 kb away from the next protein coding gene (Ferritin Mitochondrial, FTMT), the index SNVs of the remaining three regions were located directly in a protein coding gene: tubulin polymerization promoting protein (TPPP) on 5p15.33, microtubule-associated serine/threonine kinase family member 4 (MAST4) on 5q12.3 and ATPase phospholipid transporting 10B (putative) (ATP10B) on 5q34. As the variation allocated to ATB10B was a single signal, this has to be considered with caution. The same applies to the variations on chromosomes 1p35.2 (long intergenic nonprotein coding RNA 1648, LINC01648), 2p12 (regenerating family member 1 alpha, REG1A), 3p14.2 (synaptoporin, SYNPR), and 4p16.1 (SH3 domain and tetratricopeptide repeats 1, SH3TC1). The remaining suggestive signals are localized near N-acetylated alpha-linked acidic dipeptidase-like 2 (NAALADL2) on chromosome 3q26.31, spermatogenesis-associated 19 (SPATA19) on chromosome 11q25, Down syndrome critical region 4 (DSCR4) on chromosome 21q22.13, and iduronate 2-sulfatase (IDS) on the X-chromosome (Xq28).

## Discussion

In this study we used a genome-wide approach to detect common variations associated with DBN. Among the approximately 8 million SNVs tested, only rs72665334 allocated to FGF14 on chromosome 13q33.1 remained genome-wide significant (*p* < 5 × 10^−8^). Additional suggestive associations (*p* < 1 × 10^−5^) were found for 15 LD-independent regions, the most promising of which were localized on chromosome 5q14.1 in a region containing the two overlapping genes DHFR and MSH3.

FGF14, the gene underlying the genome-wide hit, is part of the fibroblast growth factor (FGF) family subgroup of intracellular nonsecretory forms and is involved in the regulation of voltage-gated ion channels in neurons. It is prominently expressed in PCs, particularly in the axon initial segment of these cells [34] modulating the density of sodium [35, 36] and potassium channels [37] and also has a regulatory effect on calcium channels [38]. FGF14 knockdown in mouse cerebellar lysates has been shown to affect multiple kinetic parameters in PCs, which are responsible for sodium channel inactivation and thereby decrease the ability of repetitive firing [36, 39]. Also, a reduction of the spontaneous firing rate following in vivo knockdown of FGF14 in mature cerebellar PCs of wild-type mice as well as a reduced excitability of these cells in FGF14 knockout mice could be observed. Subsequently, these mice showed impairment of motor coordination and balance [40].

An impaired function of PCs due to reduced excitability is also compatible with the mode of action of the potassium channel blocker 4-aminopyridine, the drug of choice for the treatment of DBN [41, 42] which increases the excitability of PCs [43]. The same applies to the 4-aminopyridine treatment of episodic ataxia type 2 (EA2), which is also associated with DBN [44, 45].

In addition to the already known physiological functions, mutations in FGF14 have been shown to cause autosomal dominant spinocerebellar ataxia 27 (SCA27), a rare inherited neurodegenerative disorder leading to cerebellar degeneration and clinically slow progressive cerebellar ataxia, oculomotor deficits including nystagmus, low performance in education, and mental retardation [46–48].

However, as LD-dependent variations in this region are sparse and associations of them are below a suggestive threshold, this result should be considered with caution.

The top hit (rs245100) in the combined region on chromosome 5q14.1, containing the genes DHFR and MSH3, is localized in intron 4 of DHFR, and its significance was slightly below the genome-wide threshold (*p* = 5.03 × 10^−7^). DHFR is an essential enzyme in the folate metabolism, which catalyzes the reduction of dihydrofolate to tetrahydrofolate and additionally, in a less efficient reaction, the reduction of folate to dihydrofolate [49]. Folates play an important role in single carbon metabolism, such as DNA synthesis and methylation, regulation of gene expression, and synthesis of amino acids, nucleic acids, and neurotransmitters [50]. There is also evidence of pro-regenerative effects of folate in the adult CNS [51], as well as on peripheral neurons [52] which depend essentially on DHFR [53]. Blocking DHFR function by administration of the folate analogue and DHFR antagonist methotrexate (MTX) leads to neurotoxic effects in the cerebellum of newborn rats [54] as well as to the degeneration of PCs in guinea pigs, which could be rescued by application of the tetrahydrofolate derivative leucovorin [55]. This rescue effect of folate is also apparent in newborn rats, in which PC atrophy and cerebellar degenerative changes induced by treatment with valproic acid in the mothers could be markedly reduced by an additional treatment with folic acid [56]. Conceivably, an impaired DHFR function may contribute to impaired cerebellar function.

The other gene in this chromosomal region is MSH3, a part of the post-replicative DNA mismatch repair system, which is important for cell cycle regulation, apoptosis, and genome stability [57]. Additionally, MSH3 has an effect on the phenotype of Friedreich’s ataxia [58] and Huntington’s disease [59], both trinucleotide repeat disorders. The process resulting in trinucleotide repeat (TNR) expansions is not entirely understood, but MSH3 seems to provide a mutagenic role for (CTG)_n_/(CAG)_n_ repeat tracts [60], while knockdown of Msh3 blocks TNR expansion effectively in mice [61]. Also, the TNR expansion disorder SCA6 frequently accompanies DBN [15, 16]. A direct link between the TNR enhancing properties of MSH3 and the occurrence of DBN requires further investigation.

In addition to these most promising associations, another twelve regions with suggestive evidence could be identified. Five of those variants localized in or near ATP10B, LINC01648, REG1A, SH3TC1, and SYNPR were single signals. Taking also into account the relatively low *p* value, these signals are probably false positives.

The remaining 7 variants were localized in genes involved in the cytoskeleton (MAST4, TPPP), iron homeostasis (FTMT), lysosomal degradation of sulfate esters (IDS), glutamate carboxypeptidases (NAALADL2), and fertility according to their sole expression in reproductive organs (DSCR4, SPATA19). Of these, MAST4 [62], TPPP [63], FTMT [64], and IDS [65] have been linked to pathological processes in the brain.

Finally, the main limitation of this study is the small sample size. Since DBN is a very rare phenotype that has received little attention in research so far, this study provides first clues to a possible genetic background. Nevertheless, confirmation of the associated signals in independent samples is necessary.

In conclusion, a genome-wide significantly associated signal points to FGF14 as a factor that might be involved in the pathophysiology of DBN. Given the expression of FGF14 protein in PC, its involvement in the regulation of neuronal ion channels, and its impact on the excitability of PCs, together with the hypothesis of DBN being a consequence of impaired PC function, these results point to an influence of genetic variations in FGF14 on the susceptibility of to this typical cerebellar nystagmus. The second signal suggests an involvement of the folate metabolism through the association of a variation in the regulating enzyme, DHFR. Both findings provide promising candidates for DBN and also for cerebellar degeneration as its cause.

## References

[1] Massaquoi SG (2012). Physiology of clinical dysfunction of the cerebellum. Handb Clin Neurol.

[2] Valente EM, Nuovo S, Doherty D (2018). Genetics of cerebellar disorders. Handb Clin Neurol.

[3] Klockgether T (2012). Sporadic adult-onset ataxia of unknown etiology. Handb Clin Neurol.

[4] Wagner JN, Glaser M, Brandt T, Strupp M (2008). Downbeat nystagmus: aetiology and comorbidity in 117 patients. J Neurol Neurosurg Psychiatry.

[5] Leigh RJ, Zee DS (2015). The neurology of eye movements.

[6] Feil K, Strobl R, Schindler A, Krafczyk S, Goldschagg N, Frenzel C, Glaser M, Schöberl F, Zwergal A, Strupp M (2018). What is behind cerebellar vertigo and dizziness?. Cerebellum.

[7] Kalla R, Deutschlander A, Hufner K, Stephan T, Jahn K, Glasauer S, Brandt T, Strupp M (2006). Detection of floccular hypometabolism in downbeat nystagmus by fMRI. Neurology.

[8] Zee DS, Yamazaki A, Butler PH, Gücer G (1981). Effects of ablation of flocculus and paraflocculus of eye movements in primate. J Neurophysiol.

[9] Pierrot-Deseilligny C, Milea D (2005). Vertical nystagmus: clinical facts and hypotheses. Brain.

[10] Marti S, Straumann D, Glasauer S (2005). The origin of downbeat nystagmus: an asymmetry in the distribution of on-directions of vertical gaze-velocity Purkinje cells. Ann N Y Acad Sci.

[11] Bohmer A, Straumann D (1998). Pathomechanism of mammalian downbeat nystagmus due to cerebellar lesion: a simple hypothesis. Neurosci Lett.

[12] Glasauer S, Hoshi M, Buttner U (2005). Smooth pursuit in patients with downbeat nystagmus. Ann N Y Acad Sci.

[13] Glasauer S, Hoshi M, Kempermann U, Eggert T, Buttner U (2003). Three-dimensional eye position and slow phase velocity in humans with downbeat nystagmus. J Neurophysiol.

[14] Choi J-H, Seo J-D, Choi YR, Kim M-J, Shin J-H, Kim JS, Choi KD (2015). Exercise-induced downbeat nystagmus in a Korean family with a nonsense mutation in CACNA1A. Neurol Sci.

[15] Yabe I, Sasaki H, Takeichi N, Takei A, Hamada T, Fukushima K, Tashiro K (2003). Positional vertigo and macroscopic downbeat positioning nystagmus in spinocerebellar ataxia type 6 (SCA6). J Neurol.

[16] Yabe I, Matsushima M, Yoshida K, Ishikawa K, Shirai S, Takahashi I, Sasaki H (2015). Rare frequency of downbeat positioning nystagmus in spinocerebellar ataxia type 31. J Neurol Sci.

[17] Liang L, Chen T, Wu Y (2016). The electrophysiology of spinocerebellar ataxias. Neurophysiol Clin.

[18] First M, Gibbon M, Spitzer R, Williams J, Benjamin L (1997). Structured clinical interview for DSM-IV Axis II personality disorders, (SCID-II).

[19] First MB, Spitzer RL, Gibbon M, Williams JBW (1996). Structured clinical interview for DSM-IV axis I disorders, clinician version (SCID-CV).

[20] Rice JP, Reich T, Bucholz KK, Neuman RJ, Fishman R, Rochberg N (1995). Comparison of direct interview and family history diagnoses of alcohol dependence. Alcohol Clin Exp Res.

[21] Rujescu D, Hartmann AM, Giegling I, Konte B, Herrling M, Himmelein S, Strupp M (2018). Genome-wide association study in vestibular neuritis: involvement of the host factor for HSV-1 replication. Front Neurol.

[22] Drago A, Giegling I, Schafer M, Hartmann A, Konte B, Friedl M, et al. Genome-wide association study supports the role of the immunological system and of the neurodevelopmental processes in response to haloperidol treatment. Pharmacogenet Genomics. 2014;24(6):314–9. 10.1097/FPC.0000000000000052. 10.1097/FPC.0000000000000052 24751813

[23] Priebe L, Degenhardt F, Strohmaier J, Breuer R, Herms S, Witt SH, Hoffmann P, Kulbida R, Mattheisen M, Moebus S, Meyer-Lindenberg A, Walter H, Mössner R, Nenadic I, Sauer H, Rujescu D, Maier W, Rietschel M, Nöthen MM, Cichon S (2013). Copy number variants in German patients with schizophrenia. PLoS One.

[24] Need AC, Ge D, Weale ME, Maia J, Feng S, Heinzen EL, Shianna KV, Yoon W, Kasperaviciūte D, Gennarelli M, Strittmatter WJ, Bonvicini C, Rossi G, Jayathilake K, Cola PA, McEvoy J, Keefe RS, Fisher EM, St Jean PL, Giegling I, Hartmann AM, Möller HJ, Ruppert A, Fraser G, Crombie C, Middleton LT, St Clair D, Roses AD, Muglia P, Francks C, Rujescu D, Meltzer HY, Goldstein DB (2009). A genome-wide investigation of SNPs and CNVs in schizophrenia. PLoS Genet.

[25] Bramon E, Pirinen M, Strange A, Lin K, Freeman C, Bellenguez C (2014). A genome-wide association analysis of a broad psychosis phenotype identifies three loci for further investigation. Biol Psychiatry.

[26] Schizophrenia Working Group of the Psychiatric Genomics Consortium (2014). Biological insights from 108 schizophrenia-associated genetic loci. Nature.

[27] Stefansson H, Rujescu D, Cichon S, Pietilainen OP, Ingason A, Steinberg S (2008). Large recurrent microdeletions associated with schizophrenia. Nature.

[28] Lencz T, Knowles E, Davies G, Guha S, Liewald DC, Starr JM, Djurovic S, Melle I, Sundet K, Christoforou A, Reinvang I, Mukherjee S, DeRosse P, Lundervold A, Steen VM, John M, Espeseth T, Räikkönen K, Widen E, Palotie A, Eriksson JG, Giegling I, Konte B, Ikeda M, Roussos P, Giakoumaki S, Burdick KE, Payton A, Ollier W, Horan M, Donohoe G, Morris D, Corvin A, Gill M, Pendleton N, Iwata N, Darvasi A, Bitsios P, Rujescu D, Lahti J, Hellard SL, Keller MC, Andreassen OA, Deary IJ, Glahn DC, Malhotra AK (2014). Molecular genetic evidence for overlap between general cognitive ability and risk for schizophrenia: a report from the cognitive genomics consortium (COGENT). Mol Psychiatry.

[29] Galfalvy H, Haghighi F, Hodgkinson C, Goldman D, Oquendo MA, Burke A, Huang YY, Giegling I, Rujescu D, Bureau A, Turecki G, Mann JJ (2015). A genome-wide association study of suicidal behavior. Am J Med Genet B Neuropsychiatr Genet.

[30] Chang CC, Chow CC, Tellier LC, Vattikuti S, Purcell SM, Lee JJ (2015). Second-generation PLINK: rising to the challenge of larger and richer datasets. Gigascience.

[31] Price AL, Patterson NJ, Plenge RM, Weinblatt ME, Shadick NA, Reich D (2006). Principal components analysis corrects for stratification in genome-wide association studies. Nat Genet.

[32] Delaneau O, Marchini J, Zagury J-F (2011). A linear complexity phasing method for thousands of genomes. Nat Methods.

[33] Howie B, Marchini J, Stephens M (2011). Genotype imputation with thousands of genomes. G3 (Bethesda).

[34] Xiao M, Bosch MK, Nerbonne JM, Ornitz DM (2013). FGF14 localization and organization of the axon initial segment. Mol Cell Neurosci.

[35] Lou J-Y, Laezza F, Gerber BR, Xiao M, Yamada KA, Hartmann H, Craig AM, Nerbonne JM, Ornitz DM (2005). Fibroblast growth factor 14 is an intracellular modulator of voltage-gated sodium channels. J Physiol.

[36] Yan H, Pablo JL, Wang C, Pitt GS (2014). FGF14 modulates resurgent sodium current in mouse cerebellar Purkinje neurons. Elife.

[37] Pablo JL, Pitt GS (2017). FGF14 is a regulator of KCNQ2/3 channels. Proc Natl Acad Sci U S A.

[38] Yan H, Pablo JL, Pitt GS (2013). FGF14 regulates presynaptic Ca2+ channels and synaptic transmission. Cell Rep.

[39] Shakkottai VG, Xiao M, Xu L, Wong M, Nerbonne JM, Ornitz DM, Yamada KA (2009). FGF14 regulates the intrinsic excitability of cerebellar Purkinje neurons. Neurobiol Dis.

[40] Bosch MK, Carrasquillo Y, Ransdell JL, Kanakamedala A, Ornitz DM, Nerbonne JM (2015). Intracellular FGF14 (iFGF14) is required for spontaneous and evoked firing in cerebellar Purkinje neurons and for motor coordination and balance. J Neurosci.

[41] Strupp M, Schüler O, Krafczyk S, Jahn K, Schautzer F, Büttner U, Brandt T (2003). Treatment of downbeat nystagmus with 3,4-diaminopyridine: a placebo-controlled study. Neurology.

[42] Strupp M, Teufel J, Zwergal A, Schniepp R, Khodakhah K, Feil K (2017). Aminopyridines for the treatment of neurologic disorders. Neurol Clin Pract.

[43] Etzion Y, Grossman Y (2001). Highly 4-aminopyridine sensitive delayed rectifier current modulates the excitability of guinea pig cerebellar Purkinje cells. Exp Brain Res.

[44] Strupp M, Kalla R, Claassen J, Adrion C, Mansmann U, Klopstock T, Freilinger T, Neugebauer H, Spiegel R, Dichgans M, Lehmann-Horn F, Jurkat-Rott K, Brandt T, Jen JC, Jahn K (2011). A randomized trial of 4-aminopyridine in EA2 and related familial episodic ataxias. Neurology.

[45] Zesiewicz TA, Wilmot G, Kuo S-H, Perlman S, Greenstein PE, Ying SH, et al. Comprehensive systematic review summary: treatment of cerebellar motor dysfunction and ataxia: report of the guideline development, dissemination, and implementation subcommittee of the American Academy of Neurology. Neurology. 2018. 10.1212/WNL.0000000000005055. 10.1212/WNL.0000000000005055 PMC5863491 29440566

[46] Brusse E, de Koning I, Maat-Kievit A, Oostra BA, Heutink P, van Swieten JC (2006). Spinocerebellar ataxia associated with a mutation in the fibroblast growth factor 14 gene (SCA27): a new phenotype. Mov Disord.

[47] Misceo D, Fannemel M, Barøy T, Roberto R, Tvedt B, Jaeger T, Bryn V, Strømme P, Frengen E (2009). SCA27 caused by a chromosome translocation: further delineation of the phenotype. Neurogenetics.

[48] Coebergh JA, van de Fransen Putte DE, Snoeck IN, Ruivenkamp C, van Haeringen A, Smit LM (2014). A new variable phenotype in spinocerebellar ataxia 27 (SCA 27) caused by a deletion in the FGF14 gene. Eur J Paediatr Neurol.

[49] Bailey SW, Ayling JE (2009). The extremely slow and variable activity of dihydrofolate reductase in human liver and its implications for high folic acid intake. Proc Natl Acad Sci U S A.

[50] Nazki FH, Sameer AS, Ganaie BA (2014). Folate: metabolism, genes, polymorphisms and the associated diseases. Gene.

[51] Iskandar BJ, Nelson A, Resnick D, Skene JH, Gao P, Johnson C, Cook TD, Hariharan N (2004). Folic acid supplementation enhances repair of the adult central nervous system. Ann Neurol.

[52] Harma A, Sahin MS, Zorludemir S (2015). Effects of intraperitoneally administered folic acid on the healing of repaired tibial nerves in rats. J Reconstr Microsurg.

[53] Iskandar BJ, Rizk E, Meier B, Hariharan N, Bottiglieri T, Finnell RH, Jarrard DF, Banerjee RV, Skene JH, Nelson A, Patel N, Gherasim C, Simon K, Cook TD, Hogan KJ (2010). Folate regulation of axonal regeneration in the rodent central nervous system through DNA methylation. J Clin Invest.

[54] Sugiyama A, Sun J, Ueda K, Furukawa S, Takeuchi T (2015). Effect of methotrexate on cerebellar development in infant rats. J Vet Med Sci.

[55] el-Badawi MG, Fatani JA, Bahakim H, Abdalla MA (1990). Light and electron microscopic observations on the cerebellum of guinea pigs following low-dose methotrexate. Exp Mol Pathol.

[56] Shona SI, Rizk AA, El Sadik AO, Emam HY, Ali EN. Effect of valproic acid administration during pregnancy on postnatal development of cerebellar cortex and the possible protective role of folic acid. Folia Morphol (Warsz). 2017. 10.5603/FM.a2017.0100. 10.5603/FM.a2017.0100 29064543

[57] Palombo F, Iaccarino I, Nakajima E, Ikejima M, Shimada T, Jiricny J (1996). hMutSbeta, a heterodimer of hMSH2 and hMSH3, binds to insertion/deletion loops in DNA. Curr Biol.

[58] Ezzatizadeh V, Sandi C, Sandi M, Anjomani-Virmouni S, Al-Mahdawi S, Pook MA (2014). MutLalpha heterodimers modify the molecular phenotype of Friedreich ataxia. PLoS One.

[59] Tomé S, Manley K, Simard JP, Clark GW, Slean MM, Swami M, Shelbourne PF, Tillier ER, Monckton DG, Messer A, Pearson CE (2013). MSH3 polymorphisms and protein levels affect CAG repeat instability in Huntington’s disease mice. PLoS Genet.

[60] López Castel A, Cleary JD, Pearson CE (2010). Repeat instability as the basis for human diseases and as a potential target for therapy. Nat Rev Mol Cell Biol.

[61] Gannon A-MM, Frizzell A, Healy E, Lahue RS (2012). MutSβ and histone deacetylase complexes promote expansions of trinucleotide repeats in human cells. Nucleic Acids Res.

[62] Martins-de-Souza D, Guest PC, Mann DM, Roeber S, Rahmoune H, Bauder C, Kretzschmar H, Volk B, Baborie A, Bahn S (2012). Proteomic analysis identifies dysfunction in cellular transport, energy, and protein metabolism in different brain regions of atypical frontotemporal lobar degeneration. J Proteome Res.

[63] Oláh J, Szénási T, Szunyogh S, Szabó A, Lehotzky A, Ovádi J (2017). Further evidence for microtubule-independent dimerization of TPPP/p25. Sci Rep.

[64] Gao G, Chang Y-Z (2014). Mitochondrial ferritin in the regulation of brain iron homeostasis and neurodegenerative diseases. Front Pharmacol.

[65] Holmes RS (2017). Comparative studies of vertebrate iduronate 2-sulfatase (IDS) genes and proteins: evolution of a mammalian X-linked gene. 3 Biotech.

